# Functional and Angiographic Outcomes Following Surgical Revascularization in Pediatric Moyamoya Disease: A Single-Centre Cohort Study from Central Asia

**DOI:** 10.3390/children13070865

**Published:** 2026-06-29

**Authors:** Chingiz Nurimanov, Karashash Menlibayeva, Iroda Mammadinova, Ainur Turzhanova, Seitzhan Aidarov, Daultay Batyrkhanov, Assel Kabykenova, Yerbol Makhambetov, David Pochivalov, Dmitriy Surdin, Serik Akshulakov, Assylbek Kaliyev

**Affiliations:** 1 Department of Vascular and Functional Neurosurgery, National Centre for Neurosurgery, Astana 010000, Kazakhstan; 2 Department of Population Health Sciences, King’s College London, London WC2R 2LS, UK; 3 Scientific Research Management Department, National Centre for Neurosurgery, Astana 010000, Kazakhstan; 4 Department of Radiology and Radiosurgery, National Centre for Neurosurgery, Astana 010000, Kazakhstan; 5 Hospital Management Sector, National Centre for Neurosurgery, Astana 010000, Kazakhstan; 6 Department of Pediatric Neurosurgery, National Centre for Neurosurgery, Astana 010000, Kazakhstan

**Keywords:** stroke, cerebral ischemia, revascularization, Moyamoya disease

## Abstract

**Highlights:**

**What are the main findings?**

- Surgical revascularization in pediatric moyamoya disease was associated with significant functional improvement and a low rate of recurrent cerebrovascular events.
- Favourable postoperative collateral formation (Matsushima grade A–B) was strongly associated with improved neurological outcomes, whereas poor collateralization was linked to worse functional recovery.

**What are the implications of the main findings?**

- The quality of postoperative revascularization may be more important for clinical outcome than the specific surgical technique used.
- These findings support surgical revascularization as an effective treatment strategy for pediatric moyamoya disease and provide novel data from an underrepresented Central Asian population.

**Abstract:**

Background: Moyamoya disease (MMD) is a progressive cerebrovascular disorder and an important cause of ischemic stroke in children. Surgical revascularization is the mainstay of treatment; however, data on functional and angiographic outcomes in pediatric populations, particularly from Central Asia, remain limited. Methods: We conducted a retrospective single-centre cohort study of 45 pediatric patients with MMD who underwent surgical revascularization between 2013 and 2025. Clinical outcomes were assessed using the modified Rankin Scale (mRS), and angiographic outcomes were evaluated using the Matsushima grading system. Associations between clinical, surgical, and radiological factors and functional outcomes were analysed. Results: The mean age at diagnosis was 9.5 ± 3.8 years, with a mean follow-up of 20.6 ± 17.5 months. Headache was the most common presenting symptom (77.8%), while ischemic stroke was the predominant cerebrovascular presentation, occurring in more than 70% of patients. More than 75% of patients had advanced angiographic disease (Suzuki stages IV–V). Functional outcomes improved significantly following surgery, with mean mRS decreasing from 2.09 ± 1.26 to 1.64 ± 1.00 (*p* = 0.004). At follow-up, 82.2% of patients achieved favorable outcomes (mRS 1–2). Bilateral revascularization was performed in 64.4% of cases. No significant differences were observed between direct, indirect, and combined techniques, although a trend toward improved outcomes with direct and combined approaches was noted. Postoperative collateral formation was strongly associated with functional outcome (*p* = 0.005). Conclusions: Surgical revascularization is associated with significant functional improvement and low recurrence rates in pediatric MMD. The quality of collateral formation, rather than the surgical technique itself, appears to be the primary determinant of outcome.

## 1. Introduction

Moyamoya disease (MMD) is an uncommon, progressive cerebrovascular disorder of unknown etiology, characterized by progressive stenosis and subsequent occlusion of the terminal internal carotid arteries (ICA) and the proximal segments of the anterior and middle cerebral arteries. This process leads to the formation of fragile compensatory collateral vessels at the base of the brain, producing the characteristic “puff-of-smoke” appearance on cerebral angiography, first described by Suzuki and Takaku in 1969 [1].

Moyamoya disease accounts for around 6-10% of pediatric cases of ischemic stroke and transient ischemic attack (TIA) [2,3]. If left untreated, it can result in severe and permanent neurological disability. In pediatric cases, the clinical course is primarily ischemic, commonly manifesting as transient ischemic attacks, hemiparesis, or seizures. Furthermore, children often experience a progressive deterioration in cognitive function [4,5]. In contrast, the clinical profile shifts significantly with age; intracranial hemorrhage is statistically more frequent in adult patients [6].

The epidemiology of MMD demonstrates marked geographic and ethnic variation, with the highest incidence reported in East Asian populations, particularly in Japan and South Korea, and represents one of the most common cerebrovascular disorders in children [7,8]. Genetic susceptibility is believed to play an important role in disease pathogenesis, most notably through variants in the RNF213 gene, which have been strongly associated with MMD in Asian populations [9,10]. Although most cases are sporadic, familial clustering has been reported, and MMD is also associated with several conditions, including Down syndrome, neurofibromatosis type 1, and sickle cell disease [10].

Surgical revascularization remains the cornerstone of treatment, aiming to improve cerebral blood flow and reduce the risk of ischemic and hemorrhagic events [11]. Current surgical strategies include direct, indirect, and combined revascularization techniques. Although all approaches have demonstrated clinical benefit, their comparative effectiveness in pediatric populations remains incompletely understood, particularly regarding long-term functional and angiographic outcomes. 

In addition, although postoperative collateral formation is a key determinant of cerebral perfusion, the relationship between angiographic revascularization (e.g., Matsushima grade) and clinical outcomes has not been fully elucidated.

In this study, we evaluated functional and angiographic outcomes in a cohort of pediatric patients with moyamoya disease undergoing surgical revascularization at a tertiary neurosurgical centre in Kazakhstan. Specifically, we aimed to assess the association between postoperative collateral formation and functional recovery, and to identify clinical and surgical factors associated with outcome. This study represents, to our knowledge, the first report describing pediatric moyamoya disease from Central Asia.

## 2. Materials and Methods

### 2.1. Study Design and Participants

This retrospective single-centre cohort study included pediatric patients diagnosed with moyamoya disease who received treatment at the National Centre for Neurosurgery (NCN) in Astana, Kazakhstan, from August 2013 to October 2025. The diagnosis of MMD was established according to the updated 2021 Diagnostic Criteria for Moyamoya Disease proposed by the Research Committee on Moyamoya Disease (Spontaneous Occlusion of the Circle of Willis) [12]. Patients older than 18 years and those with quasi-moyamoya disease (secondary moyamoya syndrome) were excluded. After applying the eligibility criteria, a total of 45 pediatric patients were included in the final analysis. 

Data were retrospectively collected from medical records, operative reports, and neuroimaging studies. Collected variables included patient demographics (age, age at the time of surgery, and sex), clinical presentation, comorbid conditions, history of stroke, and mRS score at admission.

MMD was diagnosed using magnetic resonance imaging (MRI) and digital subtraction angiography (DSA) with a biplane system (Artis Zee Biplane; Siemens, Erlangen, Germany). A multidisciplinary team of neuroradiologists and neurosurgeons independently reviewed the imaging, using the Suzuki staging system to determine the severity of MMD. 

### 2.2. Treatment

All patients in our study received microsurgical cerebral revascularization. Antiplatelet therapy (aspirin, dosed according to body weight) was administered before and after surgery and continued long-term. Three different surgical approaches were tailored: direct, indirect, and combined revascularization. Direct revascularization performed an end-to-side anastomosis between the superficial temporal artery (STA) and the M3 or M4 branches of the middle cerebral artery (MCA), ensuring accuracy via ICG videoangiography or the Faucet technique as previously described [13]. Indirect revascularization focused on encephalodurosynangiosis, while the combined method merged both techniques into one procedure. The selection of the surgical technique was individualized for each patient based on age, the caliber and suitability of the STA, clinical presentation, and the operating surgeon’s judgment.

### 2.3. Follow-Up and Outcomes

The follow-up period ranged from 6 to 79 months. Follow-up assessments were conducted by neurologists, and outcomes were reviewed by a multidisciplinary team. Clinical outcomes included the occurrence of new ischemic or hemorrhagic events and functional status measured using the mRS. Functional outcomes were categorized as favourable (mRS 1–2), moderate disability (mRS 3–4), or poor outcome (mRS 5–6). 

Radiological outcomes included evaluation of postoperative collateral formation after indirect and combined revascularization using the Matsushima grading system, as well as assessment of cerebral perfusion improvement when available. Postoperative angiographic revascularization was assessed on a hemisphere-level basis whenever follow-up angiography was available. In patients who underwent bilateral revascularization, each operated hemisphere was graded separately to account for potential differences in collateral development between sides. Additional endpoints included postoperative complications, recurrent cerebrovascular events, and the need for repeat revascularization (second surgery). 

### 2.4. Statistical Analysis

All data were collected, coded, and anonymized using Microsoft Excel. Statistical analyses were performed using IBM SPSS (version 26) and R (version 4.2.3), with all results independently verified to ensure accuracy. We summarized demographic and clinical data using descriptive statistics, expressing continuous variables as mean ± standard deviation and categorical data as frequencies and percentages. To determine our analytical approach, we used the Shapiro–Wilk test to check for normality. For comparisons between the three outcome groups—favourable, moderate disability, and poor—we utilized the Kruskal–Wallis test for non-normal data, while Chi-square or Fisher’s exact tests were applied to categorical variables as appropriate. The Wilcoxon signed-rank test was used for paired comparisons of pre- and post-operative mRS scores. This study was reported in accordance with the Strengthening the Reporting of Observational Studies in Epidemiology (STROBE) guidelines for observational studies. Data completeness was evaluated before analysis. Missing data were limited and were addressed using complete-case analysis; therefore, no imputation methods were applied. 

### 2.5. Ethical Considerations

The study was conducted in accordance with the principles of the Declaration of Helsinki. Institutional review board approval was obtained prior to data collection (Protocol No. 4, 25 September 2024), and we took great care to ensure that all legal guardians were fully informed and provided written consent for their children’s participation and follow-up.

## 3. Results

The study included 45 pediatric patients diagnosed with moyamoya disease. The average age at diagnosis was 9.5 ± 3.8 years (1.7–17.0), and the mean follow-up duration was 20.6 ± 17.5 months (range 6–79). The cohort consists of 22 boys (48.9%) and 23 girls (51.1%), demonstrating a slight female predominance, with no significant differences in sex distribution among outcome groups (Table 1).

A history of ischemic stroke was present in 32 patients (71.1%), while 2 patients (4.4%) had experienced transient ischemic attacks. There was no significant association between prior cerebrovascular history and functional outcome (Fisher’s exact test, *p* = 0.838) (Table 2), although patients with a history of ischemic stroke showed a marginally higher incidence of adverse outcomes.

At baseline, the mean modified mRS score was 2.09 ± 1.26, which improved to 1.64 ± 1.00 at follow-up. This improvement was statistically significant (Wilcoxon signed-rank test: Z = −2.862, *p* = 0.004), indicating a meaningful enhancement in functional status following surgical revascularization (Table 1).

Angiographic evaluation revealed advanced disease in the majority of patients, with a mean Suzuki stage of 4.11 ± 0.89. Stage V was the most common (40.0%), followed by Stage IV (35.6%), Stage III (20.0%), and Stage II (4.4%).

The most common presenting symptoms were headache (77.8%) and limb paresis (55.6%), followed by speech disturbances (20.0%) and seizures (15.6%). The clinical presentation demonstrated no significant differences across outcome groups (all *p* > 0.05).

A total of 74 surgical procedures were performed, demonstrating the use of staged and bilateral approaches. Bilateral revascularization was more common (64.4%) than unilateral procedures (35.6%). Direct revascularization made up 43.3% of the procedures, followed by combined techniques (36.5%) and indirect revascularization (20.3%).

The type of surgical approach (direct, indirect, or combined) did not have a significant effect on the functional outcome (Fisher’s exact test, *p* = 0.613) and was also not associated with the degree of collateral formation (*p* = 0.125), although direct bypass showed a trend toward higher rates of favourable revascularization.

During follow-up, two patients (4.4%) had recurrent cerebrovascular events. One patient developed an ischemic stroke 2 years post-surgery and was not receiving aspirin therapy at the time of the incident. The second patient, aged 17 years, experienced a hemorrhagic stroke 6 years after bilateral revascularization.

There were two postoperative surgical complications in our cohort, both involving wound-healing issues after combined bypass using both STA branches. Both resolved completely with conservative management within 21 days.

At follow-up, 82.2% of patients achieved favourable outcomes (mRS 1–2), while 15.6% had moderate disability (mRS 3–4) and 2.2% had poor outcomes (mRS 5–6).

**Figure 1:**
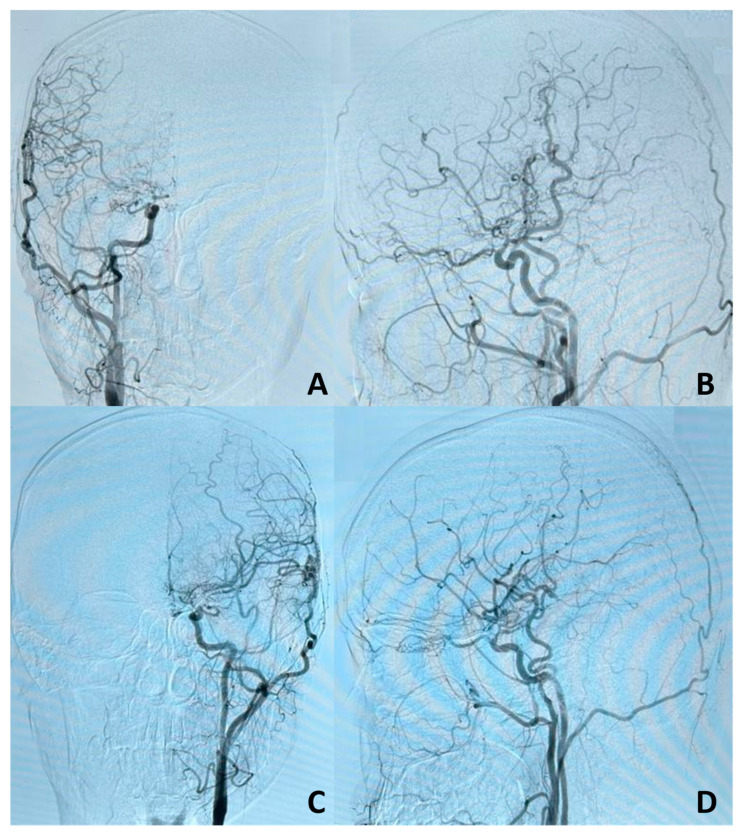
Follow-up DSA of a patient with bilateral Suzuki stage V moyamoya disease after bilateral combined revascularization. Angiography at 79 months (**A**–**D**) demonstrates excellent bilateral collateral formation (Matsushima grade A) supplied by both direct and indirect bypasses.

**Figure 2:**
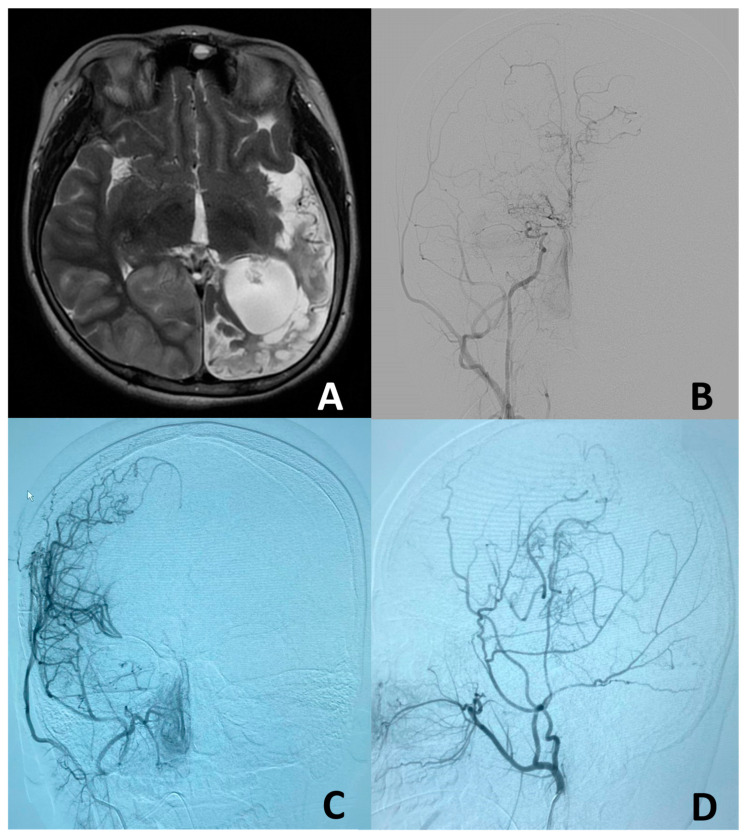
A 9-year-old girl with right-sided hemiparesis and prior left ICA territory infarction. (**A**) MRI demonstrating postischemic gliosis. (**B**) Preoperative DSA showing right terminal ICA occlusion consistent with Suzuki stage V moyamoya disease. Follow-up angiography at 14 months (**C**,**D**) demonstrates Matsushima grade B collateral formation after indirect revascularization.

**Figure 3:**
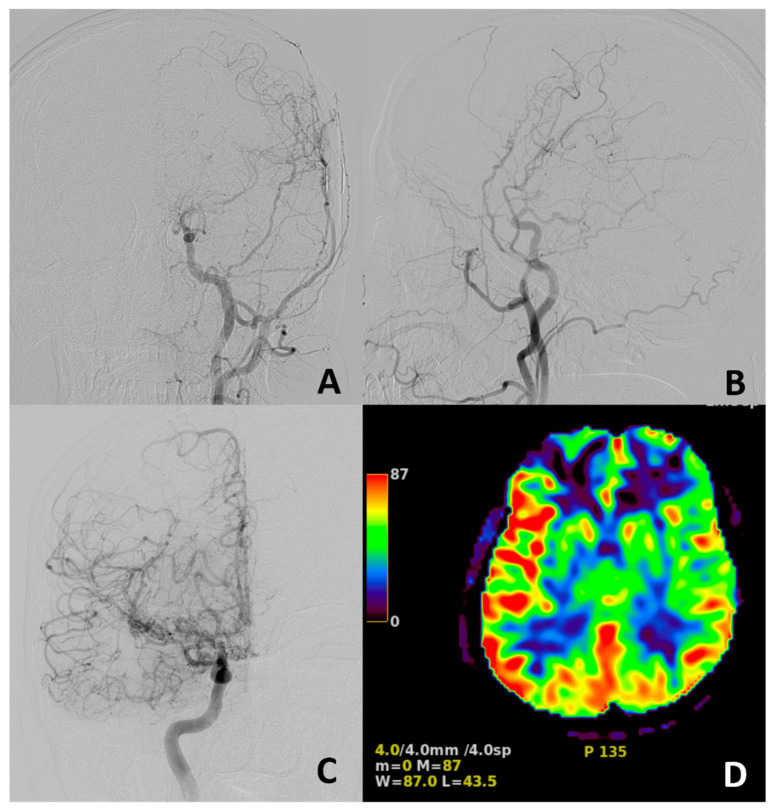
A 13-year-old girl with left-sided moyamoya disease treated with indirect revascularization. Follow-up DSA at 12 months (**A**,**B**) demonstrates Matsushima grade C collateral formation. (**C**) Right-sided Suzuki stage III disease. (**D**) ASL perfusion MRI demonstrating reduced cerebral blood flow in the left hemisphere.

Postoperative collateral formation following indirect or combined bypass was predominantly favourable, with Matsushima Grade A (Figure 1) observed in 16 patients (38.1%), Grade B (Figure 2) in 21 patients (50.0%), and Grade C (Figure 3) in 5 patients (11.9%) (Table 2). Importantly, Matsushima grade was strongly associated with functional outcome, with poorer collateral formation linked to worse mRS scores (Fisher’s exact test, *p* = 0.005). This relationship was further supported by a significant association between Matsushima grade and functional outcome at follow-up.

## 4. Discussion

This single-centre pediatric cohort study demonstrates that surgical revascularization is associated with significant functional improvement and a low rate of recurrent cerebrovascular events, even among patients presenting with advanced angiographic disease. Although Moyamoya disease predominantly affects Asian populations, data from Central Asia have not previously been reported. To our knowledge, this is the first study to describe childhood-onset Moyamoya disease from this region. 

In agreement with previous studies [3], ischemic stroke represents the most common clinical presentation of moyamoya disease in children, with reported rates ranging from 31% to 68% [14]. In our cohort, the ischemic manifestations occurred in more than 70% of patients. This observation reinforces the established distinction between pediatric and adult moyamoya disease, where ischemic manifestations predominate in children, while hemorrhagic presentation is more commonly observed in adults. More than three-quarters of our cohort presented with advanced angiographic disease (Suzuki stages IV–V), suggesting delayed diagnosis and substantial hemodynamic compromise before treatment. Despite this advanced disease burden, collateral circulation scores were not associated with baseline neurological status or presenting symptoms, indicating that angiographic severity alone may not fully reflect clinical presentation. Similar observations have been reported previously [15].

In addition to ischemic stroke, other clinical manifestations were frequently observed. The most common presenting symptom was headache, which was seen in 77.8% cases, while seizures were seen in 15.6% cases. Headaches in moyamoya disease are thought to represent chronic cerebral hypoperfusion and altered vascular reactivity [16], while seizures may occur secondarily to cortical ischemia or prior infarction [17]. Both symptoms are clinically relevant, but they did not show a significant association with functional status at presentation or outcome, further emphasizing the complex and heterogeneous clinical expression of the disease.

The predominance of bilateral disease in our series reflects the progressive nature of pediatric moyamoya disease. Nearly two-thirds of patients underwent bilateral revascularization, whereas unilateral surgery was performed in approximately one-third, reflecting either unilateral disease at presentation or staged treatment strategies. Previous evidence suggests that unilateral moyamoya disease in children frequently progresses to bilateral involvement, supporting the need for careful longitudinal surveillance following diagnosis [18,19]. 

Our findings demonstrate that surgical revascularization confers a meaningful clinical benefit in pediatric moyamoya disease, as evidenced by a statistically significant improvement in functional status and a high proportion of favorable outcomes at follow-up. Specifically, mean mRS scores improved from 2.09 ± 1.26 at baseline to 1.64 ± 1.00 postoperatively, with 82.2% of patients achieving favorable functional outcomes (mRS 1–2). These results are consistent with the existing literature, which shows that revascularization enhances cerebral perfusion, reduces the risk of recurrent ischemic events, and contributes to stabilization or improvement of neurological function in pediatric populations [15,20]. Another factor which may contribute to these favorable results is the fact that in general pediatric patients have a higher potential to respond to surgical intervention compared with adults [21].

A key finding of this study was the association between postoperative collateral formation and functional recovery. Patients with favorable angiographic revascularization (Matsushima grades A–B) were substantially more likely to achieve good neurological outcomes, whereas poor collateral development (Grade C) was associated with greater disability. This observation suggests that the effectiveness and durability of cerebral revascularization may be more important determinants of long-term outcome than the surgical technique itself [22,23].

Nevertheless, the interpretation of Matsushima grading warrants caution. The Matsushima grading system was originally designed to assess indirect collateral formation [24], and therefore primarily reflects the angiogenic contribution of indirect or combined procedures. In cases of direct bypass, where flow augmentation is immediate and does not depend on neovascularization, Matsushima grading does not fully capture the hemodynamic benefit of the procedure. Consequently, the observed association between Matsushima grade and outcome in our study likely reflects the effectiveness of indirect collateralization, particularly in patients undergoing combined revascularization. This distinction is critical when interpreting angiographic outcomes and comparing surgical strategies.

Although no statistically significant differences in functional outcomes were observed between direct, indirect, and combined revascularization techniques in our cohort, a trend toward more favorable outcomes with direct and combined approaches was noted. In our series, direct bypass accounted for 43.3% of procedures and combined techniques for 36.5%, with both approaches demonstrating higher rates of favorable collateral formation and functional recovery, although these differences did not reach statistical significance.

These findings are consistent with recent systematic review and meta-analytic evidence indicating that surgical revascularization yields superior clinical outcomes compared with conservative management, while demonstrating comparable effectiveness between direct/combined and indirect techniques [25]. Similarly, Zheng et al. reported that combined, direct, and indirect bypass procedures had comparable effects on long-term clinical outcomes in pediatric moyamoya disease, whereas surgical revascularization overall was superior to conservative treatment [26]. The benefit of surgical flow augmentation, regardless of technique, appears to be particularly relevant in pediatric patients with symptomatic moyamoya disease [27].

The low rate of recurrent cerebrovascular events observed in our cohort further supports the effectiveness of surgical revascularization. None of the patients were receiving antiplatelet therapy before referral, suggesting delayed recognition and limited awareness of moyamoya disease in the regional healthcare setting. In addition, one recurrent ischemic event occurred in a patient who had discontinued aspirin therapy, emphasizing the importance of postoperative medical management as an adjunct to surgical treatment [28]. 

Several limitations should be acknowledged in this study. The retrospective design and relatively small sample size limit the statistical power, particularly the very small poor-outcome subgroup (*n* = 1) and the generalizability of the findings. The single-centre setting may introduce selection bias and reflect institutional expertise. In addition, the follow-up duration, although sufficient for assessing short- to mid-term outcomes, does not allow for comprehensive evaluation of long-term durability, late stroke risk, or cognitive outcomes. Finally, the lack of standardized quantitative perfusion imaging limits the ability to directly correlate angiographic findings with hemodynamic improvement. A methodological limitation of this study is that functional outcome was assessed at the patient level using mRS, whereas angiographic revascularization was evaluated at the hemisphere level. This discrepancy may not fully capture the complexity of bilateral disease, where collateral formation can differ between hemispheres.

Despite these limitations, this study provides novel data on pediatric moyamoya disease from an underrepresented region and highlights the relationship between postoperative collateral formation and neurological recovery. Future prospective multicentre studies incorporating standardized perfusion imaging, neurocognitive assessment, and longer follow-up are needed to better define predictors of durable clinical outcomes and optimal surgical strategies.

## 5. Conclusions

Surgical revascularization in pediatric moyamoya disease was associated with significant functional improvement and a low rate of recurrent cerebrovascular events, even in patients presenting with advanced angiographic disease. Favorable postoperative collateral formation was strongly associated with improved neurological outcomes, suggesting that the quality of revascularization may be more important than the specific surgical technique itself. These findings support surgical revascularization as an effective treatment strategy for pediatric moyamoya disease and provide novel data from an underrepresented Central Asian population.

## Figures and Tables

**Table 1:** Characteristics of the study population and surgical procedures.

Characteristics	No of Patients/Procedure (%)
Baseline characteristics (*n* = 45 patients)	
Age at diagnosis, years (mean ± SD)	9.5 ± 3.8 (1.7–17.0)
Female:male ratio, no (%)	23 (51.1)/22 (48.9)
Follow-up, months (mean ± SD)	20.6 ± 17.5 (6–79)
mRS before (mean ± SD)	2.09 ± 1.26
mRS after (mean ± SD)	1.64 ± 1.00
Suzuki stage (mean ± SD)	4.11 ± 0.89
Radiological evaluation (*n* = 42)	
Matsushima Grade A	16 (38.1)
Matsushima Grade B	21 (50.0)
Matsushima Grade C	5 (11.9)
Clinical manifestation (*n* = 45)	
Headache, *n* (%)	35 (77.8)
Limb paresis, *n* (%)	25 (55.6)
Seizures, *n* (%)	7 (15.6)
Speech disturbances, *n* (%)	9 (20.0)
Cognitive impairment, *n* (%)	3 (6.7)
Surgical details (*n* = 74)	
One side surgery	16 (35.6)
Bilateral surgery	29 (64.4)
Direct bypass	32 (43.3)
Indirect bypass	15 (20.3)
Combined bypass	27 (36.5)
Clinical outcome	
Recurrent stroke	2 (4.4)
Postoperative wound complications	2 (4.4)

**Table 2:** Factors associated with functional outcomes.

Variable	Favorable (*n* = 37)	Moderate (*n* = 7)	Poor (*n* = 1)	*p*-Value
Age, years (mean ± SD)	9.82 ± 3.79	8.43 ± 3.69	5.0	0.62
Sex				
Male, *n* (%)	18 (81.8%)	3 (13.6%)	1 (4.5%)	0.98
Female, *n* (%)	19 (82.6%)	4 (17.4%)	0 (0%)	
Prior cerebrovascular history				
No prior stroke	10 (90.9%)	1 (9.1%)	0 (0%)	0.838
Ischemic stroke	25 (78.1%)	6 (18.8%)	1 (3.1%)	
TIA	2 (100%)	0 (0%)	0 (0%)	
Clinical presentation				
Headache, *n* (%)	30 (85.7%)	4 (11.4%)	1 (2.9%)	0.28
Limb paresis, *n* (%)	20 (80.0%)	4 (16.0%)	1 (4.0%)	0.29
Seizures, *n* (%)	5 (71.4%)	1 (14.3%)	1 (14.3%)	0.31
Surgical characteristics				
Unilateral surgery	12 (75.0%)	3 (18.8%)	1 (6.3%)	0.48
Bilateral surgery	25 (86.2%)	4 (13.8%)	0 (0%)	
Surgical technique (procedures, *n* = 74)				
Direct bypass	28 (87.5%)	4 (12.5%)	0 (0%)	0.613
Indirect bypass	12 (80.0%)	3 (20.0%)	0 (0%)	
Combined bypass	20 (74.1%)	6 (22.2%)	1 (3.7%)	
Radiological outcome				
Matsushima Grade A	15 (93.75%)	1 (6.25%)	0 (0%)	**0.005**
Matsushima Grade B	15 (71.4%)	6 (28.6%)	0 (0%)	
Matsushima Grade C	2 (40.0%)	2 (40.0%)	1 (20.0%)	

**Note**: Bold values indicate statistically significant differences (*p* < 0.05).

## Data Availability

The underlying dataset are available from the corresponding author upon reasonable request for academic purposes due to ethical and privacy restrictions.
